# A prospective randomized clinical trial of active-fluidics versus gravity-fluidics system in phacoemulsification for age-related cataract (AGSPC)

**DOI:** 10.1080/07853890.2022.2098375

**Published:** 2022-07-15

**Authors:** Yu Luo, Hongyu Li, Wenqian Chen, Yi Gao, Tianju Ma, Zi Ye, Zhaohui Li

**Affiliations:** a Medical School of Chinese People’s Liberation Army, Beijing, China; b Department of Ophthalmology, Chinese People’s Liberation Army General Hospital, Beijing, China

**Keywords:** Cataract, phacoemulsification, active-fluidics system, gravity-fluidics system, randomized controlled trial

## Abstract

**Background:**

To figure out the efficacy, effects, safety and patient’s subjective perceptions of phacoemulsification with the active-fluidics system (AFS).

**Patients and methods:**

This was a prospective, randomized, double-masked, controlled clinical study. Age-related cataract patients were recruited and randomly assigned to the AFS group and gravity-fluidics system (GFS) group in a ratio of 1:1 to have phacoemulsification. Participants were followed up at one day, one week, one month and three months postoperatively (Chinese Clinical Trial Registry, ChiCTR2100044409).

**Results:**

The overall included participants were 107 finally. The total aspiration time of the AFS group was significantly less than that of the GFS group (*p* = .020), while no significant difference existed in cumulative dissipated energy and estimated fluid usage between the two groups. The best corrected visual acuity was significantly better in the AFS group at one day and one week postoperatively (*p* = .002, *p* = .038 respectively). The recovery of central corneal thickening and macular superficial vasculature increase was earlier in the AFS group. The central retinal thickness was significantly higher in the GFS group at one month and three months postoperatively (*p* = .029, *p* = .016 respectively). The incidence of corneal adverse events was higher in GFS group (*p* = .035). No serious adverse events occurred in either group. Pain scores and the scores of Cat-PROM5 questionnaire of the AFS group were significantly lower than that of the GFS group (*p* = .011, *p* = .002 respectively).

**Conclusion:**

AFS improves the efficiency, effects, safety and patients’ subjective perceptions of phacoemulsification compared with GFS. It is worthwhile to promote its application in cataract surgery.

**KEY MESSAGES**
- The active-fluidics system automatically detects and maintains stable intraocular pressure at the set value.
- The active-fluidics system improves the efficiency, effects, safety and patients’ subjective perceptions in phacoemulsification.

## Introduction

Phacoemulsification has become the first choice of treatment for cataract on account of its superiorities like less complications and faster recovery than extracapsular cataract extraction [1,2]. It is widely used to cure cataract, the leading cause of vision impairment around the world [3]. However, surgeons still face many challenges today, such as the instability of the anterior chamber during the operation, patients’ complaints of pain and unsatisfactory visual recovery in the early post-operative period [4,5].

For decades, phacoemulsification has been performed with the gravity-fluidics system (GFS), which relies on the height difference between the patient’s eye and the balanced salt solution (BSS) bottle to generate irrigation pressure [6,7]. By setting the bottle height, the flow rate could be adjusted upwards or downwards to meet different intraoperative demands [7,8]. However, as the rate of aspiration changes dynamically, surgeons have to halt the operation to regulate the bottle height according to the anterior chamber depth (ACD) in a timely manner [9]. This delayed modulation could lead to the instantaneous intraocular pressure (IOP) fluctuations, which adversely affects the retinal blood circulation and needs to be improved [10,11].

This situation changed when the active-fluidics system (AFS) debuted [12], because it theoretically allows the pre-setting of an IOP value and maintains the target IOP through regulating the irrigation pressure [9,13]. It has been concluded that AFS is effective in maintaining the target IOP intraoperatively with better anterior chamber stability and smaller surge in laboratory studies [9,14]. Several clinical studies have compared the efficiency between AFS and GFS, but there are controversies. Solomon *et al.* and Gonzalez-Salinas *et al.* concluded that AFS improved the efficiency of phacoemulsification and saved the cumulative dissipated energy (CDE), but Malik and colleagues reported that no significant difference existed in CDE between the two systems with the same phaco tip [13,15,16]. Also, results are not entirely consistent across studies, with the conservation of CDE associated with AFS varied from 13.5% to 40% [13,15,17,18]. However, most studies are conducted on different phacoemulsifiers, which might cause potential bias and reduce the credibility of the results [14,15,17]. A similar situation occurs in the reports on other surgical outcomes such as corneal endothelial cells, postoperative IOP and the retinal vasculature [18–20]. In addition, few clinical outcomes were published as high-level evidence.

AGSPC is a randomized clinical trial with the aim of comprehensively evaluating the results of phacoemulsification with AFS and what the new system changes compared with the conventional GFS. It is anticipated to inform the future application of AFS and provide reliable evidence for improving cataract surgery strategy.

## Patients and methods

### Trial design

**Figure 1.**
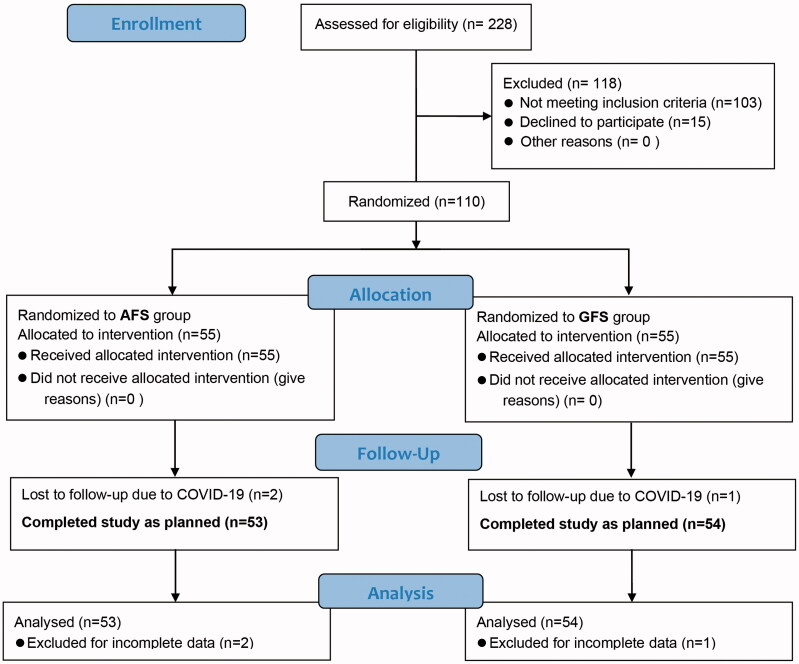
Study enrolment, allocation, follow-up and analysis flow chart.

The AGSPC study was a prospective, double-masked, single-centre randomized controlled clinical trial (Chinese Clinical Trial Registry, ChiCTR2100044409). Patients diagnosed with age-related cataract were recruited to have phacoemulsification with AFS and GFS, respectively. The follow-ups were performed at one day, one week, one month and three months postoperatively as the flow chart shown in Figure 1. The whole study was carried out in a tertiary hospital, the Chinese People's Liberation Army (PLA) General Hospital (Beijing, China). The Declaration of Helsinki was adhered, and each participant had the right to withdraw at any time. The study protocol had been approved by the Ethics Committee of the PLA General Hospital (No. S2021-068-01) and published [21].

### Study participants

Recruitment took place at the outpatient department of the PLA General Hospital. A designated ophthalmologist explained the study protocol to every potential participant in detail, and it was up to the patients themselves to decide whether to participate in or not. Whichever the choice was made, the treatment process was not affected.

Patients who met all the following criteria were eligible to be recruited [21]: (1) age-related cataract patients, whose nuclear colour (NC) and nuclear opalescence (NO) were scored as 2.0–4.9 according to The Lens Opacities Classification System III (LOCS III) [22]; (2) the best corrected visual acuity (BCVA) was better than 0.1 (Snellen equivalent 20/200) preoperatively; (3) aged between 50 and 90 years; (4) with good health, no intraocular surgery history; (5) informed consent was signed by the participant who was capable of accomplishing the whole follow-up process; (6) all examinations before the operation were done with enough quality; (7) phacoemulsification was successfully performed without conversion to other surgical methods due to intraoperative adverse events; (8) no history of long-term ocular medication use.

Any of the following would be excluded from the study [21]: (1) unable to undergo the cataract surgery with good cooperation; (2) the correlation between previous history of trauma or surgery and the lesion of the lens could not be ruled out; (3) the combination of other eye diseases that might affect BCVA or ocular blood circulation, such as corneal disease, glaucoma, uveitis, endophthalmitis, macular degeneration, diabetic retinopathy, retinal vascular obstruction, retinal detachment, etc.; (4) incomplete follow-up information, with more than one missing visit; (5) participating in other clinical trials.

### Randomization and masking

An independent researcher was responsible for the entire randomization and confidentiality. The randomization sequence list was obtained from the randomization website (www.sealedenvelope.com). Patients were randomly assigned to the AFS group or GFS group in a 1:1 ratio according to their order of enrolment.

The study was double-masked, meaning that neither the patients nor the data analysts were aware of the allocation. In addition, the doctor in charge of follow-ups was also masked. The unblinding was carried out on the completion of the whole trial because no serious complication that threatens the participants' vision or life occurred. More details have been stated in the published protocol.

### Perioperative interventions

All the participants had preoperative ophthalmic examinations including slit-lamp biomicroscopy, visual acuity, IOP measurement, fundoscopy, B-scan ultrasound and biometry measurement. The first Cat-PROM 5 questionnaire was also completed during this period [23]. Participants of the AFS group had phacoemulsification with the AFS of CENTURION^®^ Vision System (Centurion^®^) (Alcon Laboratories, Texas, USA), and the target IOP was set at 50 mmHg. The GFS group participants had the same operation with the GFS of Centurion^®^, and the bottle height was put at 90 cm. The vacuum level and aspiration flow rate were 450 mmHg and 45 cc/min respectively in both groups. The Intrepid balanced tip was applied to the two groups and the same ophthalmic surgeon performed all the surgeries. The surgical procedures and prescriptions in the perioperative period were the same in both groups (see protocol for details).

### Outcomes

The results of efficiency, effects, safety and patients’ subjective perceptions of the two fluidics systems in phacoemulsification were compared. Indicators of surgical efficiency include the CDE, estimated fluid usage (EFU) and total aspiration time (TAT), while the postoperative BCVA was evaluated as the main effect. The safety outcomes include: IOP, central corneal thickness (CCT), endothelial cell density (ECD), coefficient of variation (CV), percentage of hexagonal cells (HEX), central retinal thickness (CRT), retinal nerve fibre layer (RNFL) thickness, macular superficial vasculature and the area of the foveal avascular zone (FAZ). The foveal, parafoveal, perifoveal and whole area were defined according to the ETDRS grids [24]. The Wong-Baker Faces Pain Rating Scale (WBS) [25] and the Cat-PROM 5 questionnaire were used to assess subjective perceptions. Every participant’s demographic, ophthalmic examinations and disease history information were collected preoperatively. The efficiency data along with the pain scores were recorded immediately after each operation. Other indicators were collected at each follow-up visit. Each of the examinations was performed by the same doctor, who had been trained for the study. The apparatus and methods of examination, as well as the timeline and data collection schedule, were presented in the protocol.

### Statistical analysis

Sample size calculations were based on a published randomized controlled clinical trial that compared the surgical efficiency of the two systems [18]. Their results showed that the CDE could be lowered by approximately 23% when the AFS was used. With a predicted drop-out rate of 10% at a two-sided test level of α = 0.05 and power = 0.8, a sample size of 110 was certified. In addition, we also referred to the results of another Chinese study on the effects of AFS and GFS in phacoemulsification [26]. A significant difference in BCVA between the two groups was reported at one week postoperatively. To verify this difference, we also had a calculation based on the same test level, and a sample size of 68 was qualified. There were no other studies reported similar outcomes like us at the beginning of the trial design. Therefore, the CDE and BCVA were designed to be the main outcomes, and we took the larger volume of sample as the final sample size.

All the continuous variables were shown as mean ± standard deviation (SD), while categorical variables were presented as whole numbers and percentages. Baseline characteristics were compared firstly to assess the balance between the two groups. Then, results from both groups at the same follow-up timepoint were compared to verify whether differences exist. Also, the data of both groups were compared with their own baseline. For continuous variables that conform to a normal distribution, the group *t*-test was used. The Mann–Whitney U-test was used for continuous variables that do not conform to a normal distribution, and the Chi-square test or Fisher's exact test for all categorical variables. IBM SPSS Statistics 26.0 (SPSS Inc., Chicago, IL, USA) was selected as the statistical analysis software, and all tests were two-sided, with *p* < .05 as the threshold. All the figures were created by Origin Pro 2021 (OriginLab Corporation, Northampton, MA, USA). No interim analysis was performed during the study.

## Results

### Patient dispositions

Recruitment for this trial started in March 2021 and was completed in January 2022. A total of 228 consecutive patients were assessed for eligibility, 118 patients did not meet the inclusion criteria or refused to participate, 110 patients were enrolled in the study. Due to the COVID-19 pandemic, three participants were unable to accomplish follow-up visits on time and were excluded from data analysis. Finally, the study included 107 participants, 53 in the AFS group and 54 in the GFS group (Figure 1). All patients received the correct intervention and no intentional analysis was performed. Demographic characteristics, biological measurements and disease history are presented in Table 1, indicating that the numbers of participants in the two groups were comparable in terms of age, sex, axial length (AL), ACD, NO, NC, diabetes and hypertension. All the implanted intraocular lens (IOLs) were aspherical hydrophobic acrylic but with different A constant. The appropriate IOL that best meets the target refraction was selected by the surgeon based on the biometry measurement for each participant (Supplementary Table 1).

**Table 1.** Baseline demographics and characteristics.

	AFS	GFS	*p* value
Eyes, *n*	53	54	
Age years, mean ± SD	71.35 ± 9.86	72.15 ± 9.18	.801^a^
Male, *n* (%)	31 (58.49)	23 (42.59)	.1
AL mm, mean ± SD	24.40 ± 1.74	23.75 ± 1.31	.056^a^
ACD mm, mean ± SD	3.05 ± 0.31	3.00 ± 0.34	.581^a^
NO, mean ± SD	3.25 ± 0.86	3.12 ± 0.84	.398^a^
NC, mean ± SD	3.25 ± 0.83	3.09 ± 0.86	.199^a^
HBP, *n* (%)	23 (43.40)	31 (57.41)	.147
Diabetes, *n* (%)	17 (32.76)	19 (35.19)	.734

^a^Mann–Whitney U-test.

AFS: active-fluidics system; GFS: gravity-fluidics system; SD: standard deviation; AL: axial length; ACD: anterior chamber depth; NO: nuclear opalescence; NC: nuclear colour; HBP: hypertension.

### Efficacy and effects outcomes

The statistical results showed that TAT of the AFS group was significantly less than that of the GFS group (*p* = .020) (Table 2). Although CDE was higher in the GFS group, the difference was not statistically significant (*p* = .569). A similar situation was seen with EFU, there was also no statistically significant difference between the two groups (*p* = .084).

**Table 2.** Efficacy, effects and subjective perception outcomes (mean ± SD).

	AFS	GFS	*p* Value
*Efficacy*			
CDE (percent seconds)	3.58 ± 3.09	4.08 ± 3.25	.569^a^
TAT (seconds)	143.83 ± 40.29	158.44 ± 35.84	.020^a^
EFU (ml)	45.74 ± 12.71	49.00 ± 11.45	.084^a^
*Effects*			
BCVA (LogMAR)			
preoperative	0.37 ± 0.28	0.40 ± 0.30	.603^a^
1 day	0.06 ± 0.13	0.11 ± 0.11	.002^a^
1 week	0.03 ± 0.07	0.06 ± 0.09	.038^a^
1 month	0.04 ± 0.08	0.06 ± 0.09	.338^a^
3 months	0.03 ± 0.07	0.06 ± 0.08	.153^a^
*Subjective perception*			
WBS	0.74 ± 1.80	1.30 ± 1.61	.011^a^
Cat-PROM 5 scores			
preoperative	1.24 ± 2.48	1.18 ± 1.93	.915^a^
1 month	−5.80 ± 1.38	−5.01 ± 0.70	.002^a^

^a^Mann–Whitney U-test.

AFS: active-fluidics system; GFS: gravity-fluidics system; SD: standard deviation; CDE: cumulative dissipated energy; TAT: total aspiration time; EFU: estimated fluid usage; BCVA: best corrected visual acuity; WBS: Wong-Baker Faces Pain Rating Scale.

The preoperative and postoperative BCVA are presented in Table 2. There was no significant difference in BCVA between the two groups preoperatively. However, BCVA were significantly better in the AFS group at one day and one week after the surgery (*p* = .002, *p* = .038, respectively).

### Safety outcomes

**Figure 2.**
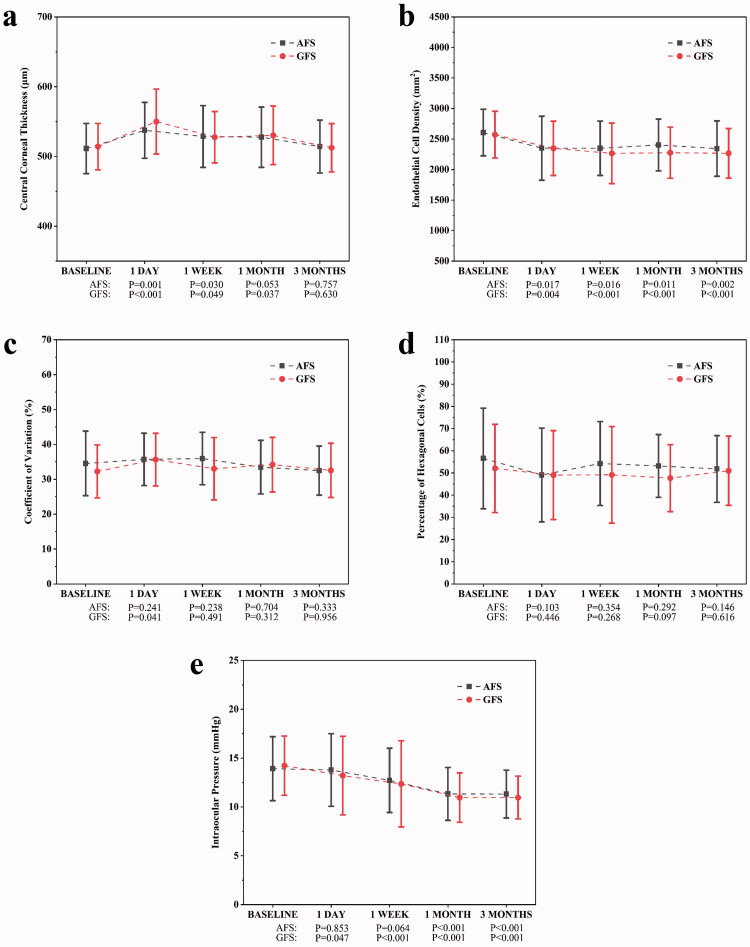
Changes in central corneal thickness (a), endothelial cell density (b), coefficient of variation (c), percentage of hexagonal cells (d) and intraocular pressure (e) after the operation. *p* values were calculated by comparing the data at each time point with the baseline. AFS: active-fluidics system, GFS: gravity-fluidics system.

There was no difference in CCT, ECD, CV, HEX and IOP between the two groups before the surgery (Supplementary Table 2). CCT increased significantly in the AFS group at one day and one week postoperatively, but returned to baseline status by one month. In the GFS group, CCT significantly increased not only at one day and one week postoperatively but also at one month, and it did not return to baseline status until three months. ECD in both groups decreased significantly after phacoemulsification, but no significant difference exists in the comparison between the two groups. The percentage of ECD loss was higher in the GFS group at three months postoperatively, although the difference was not statistically significant. CV in the GFS group significantly increased at one day postoperatively, but this change was no longer significant from one week onwards. In contrast, no significant variations occurred in CV in the AFS group. HEX did not change significantly in either group. IOP in the AFS group was not significantly altered at one day and one week postoperatively, but decreased significantly at one month and three months. However in the GFS group, IOP significantly decreased from 1 day postoperatively onwards. Changes in CCT, ECD, CV, HEX and IOP are shown in Figure 2.

The macular superficial vasculature significantly increased in both groups after the surgery (as shown in Table 3). Vessel density (VD) and perfusion density (PD) at the foveal, parafoveal, perifoveal and whole regions significantly increased in the AFS group at one week and one month, and there was no significant difference at three months postoperatively. In contrast, VD and PD of the GFS group continued to increase postoperatively at all these regions. The FAZ was not significantly changed in either group.

**Table 3.** Changes of retinal vasculature at different times (mean ± SD).

	AFS				GFS			
	1 Day	1 Week	1 Month	3 Months	1 Day	1 Week	1 Month	3 Months
*VD (inverse mm)*								
Fovea	3.45 ± 2.26	4.68 ± 2.68	5.39 ± 3.05	4.35 ± 2.80	2.51 ± 2.15	3.99 ± 2.75	4.13 ± 3.07	3.98 ± 2.76
*p value^#^*		.015^a^	<.001	.132^a^		.002^a^	.003^a^	.003^a^
Parafovea	11.85 ± 3.44	13.79 ± 3.12	14.21 ± 3.18	12.59 ± 3.62	9.58 ± 4.09	12.90 ± 3.45	12.49 ± 3.95	11.65 ± 4.25
*p value^#^*		.004^a^	<.001^a^	.390^a^		<.001	<.001	.011
Perifovea	12.86 ± 2.91	14.25 ± 3.24	14.83 ± 2.96	13.59 ± 2.85	11.12 ± 3.38	13.69 ± 3.17	13.48 ± 3.12	13.19 ± 3.79
*p value^#^*		.015^a^	.001^a^	.212^a^		<.001	<.001	.004
Whole	11.64 ± 3.45	13.88 ± 3.06	14.41 ± 2.92	13.12 ± 2.86	10.43 ± 3.55	13.24 ± 3.10	13.00 ± 3.13	12.63 ± 3.80
*p value^#^*		.001^a^	<.001^a^	.053^a^		<.001	<.001	.002
*PD (%)*								
Fovea	7.40 ± 4.78	10.06 ± 6.17	11.56 ± 6.80	9.68 ± 6.13	5.73 ± 4.29	8.87 ± 5.85	8.98 ± 6.73	8.72 ± 6.06
*p value^#^*		.028^a^	.001^a^	.083^a^		.003^a^	.010^a^	.009^a^
Parafovea	27.51 ± 8.47	31.81 ± 8.62	33.13 ± 7.97	29.10 ± 8.97	21.08 ± 9.92	29.96 ± 8.65	29.12 ± 9.95	26.88 ± 10.39
*p value^#^*		.008^a^	.001^a^	.452^a^		<.001	<.001	.004
Perifovea	30.70 ± 7.60	34.20 ± 8.31	35.70 ± 7.68	33.41 ± 7.48	26.25 ± 8.67	32.89 ± 8.30	32.35 ± 8.21	31.49 ± 9.70
*p value^#^*		.018^a^	.002^a^	.058^a^		<.001	<.001	.003
Whole	29.30 ± 7.27	33.07 ± 7.83	34.45 ± 7.54	31.97 ± 7.55	24.50 ± 8.58	31.55 ± 8.04	30.94 ± 8.14	29.79 ± 9.58
*p value^#^*		.009^a^	.001^a^	.061^a^		<.001	<.001	.003
*FAZ (mm2)*	0.17 ± 0.10	0.19 ± 0.11	0.19 ± 0.11	0.18 ± 0.10	0.18 ± 0.09	0.18 ± 0.10	0.16 ± 0.10	0.17 ± 0.11
*p value^#^*		.310^a^	.418^a^	.702		.802	.315^a^	.667^a^

^a^Mann–Whitney U-test. ^#^*p* values were calculated by comparing the data at each time point with the 1 day data.

AFS: active-fluidics system; GFS: gravity-fluidics system; SD: standard deviation; VD: vessel density; PD: perfusion density; FAZ: foveal avascular zone.

In terms of CRT and RNFL thickness, there was no significant difference between the two groups at one day postoperatively, and they were not significantly changed at one week in both groups (Table 4). However, the CRT increased significantly from one month postoperatively and it was significantly higher in the GFS group at one month and three months. Ganglion cell layer (GCL) and RNFL thickness, although not statistically different between the two groups postoperatively, increased in both groups. The increase appeared early in the GFS group (one week), whereas it was evident at one month postoperatively in the AFS group.

**Table 4.** Changes in retinal thickness (mean ± SD).

	(0) 1 day	(1) 1 week	(2) 1 month	(3) 3 months	*p* value		
					0 vs. 1	0 vs. 2	0 vs. 3
CRT (μm)							
(a) AFS	264.66 ± 13.42	269.13 ± 15.50	277.21 ± 14.81	278.38 ± 14.52	.050^a^	<.001	<.001
(b) GFS	269.74 ± 17.10	275.83 ± 17.92	285.35 ± 22.37	285.87 ± 17.16	.074	<.001	<.001
*p value^#^*	.091	.135^a^	.029	.016			
GCL (μm)							
(a) AFS	72.58 ± 10.50	75.75 ± 10.15	78.58 ± 8.35	78.87 ± 8.79	.052^a^	.002^a^	.001^a^
(b) GFS	73.22 ± 11.07	76.91 ± 12.92	78.11 ± 13.18	80.31 ± 10.25	.04^a^	.014^a^	<.001^a^
*p value^#^*	.813	.421^a^	.861^a^	.272^a^			
RNFL (μm)							
(a) AFS	87.58 ± 10.88	90.96 ± 12.3	93.30 ± 12.1	92.60 ± 12.26	.063^a^	.012	.028
(b) GFS	86.94 ± 13.84	92.44 ± 14.41	97.89 ± 15.71	96.20 ± 14.16	.046	<.001^a^	.001
*p value^#^*	.791	.805^a^	.089^a^	.163			

^a^Mann–Whitney U-test. ^#^*p* value means a vs. b.

AFS: active-fluidics system; GFS: gravity-fluidics system; SD: standard deviation; CRT: central retinal thickness; GCL: ganglion cell layer; RNFL: retinal nerve fibre layer.

The results of ocular adverse events are shown in Table 5. No serious adverse events occurred in either group. There was also no conversion to other surgical methods owing to intraoperative adverse events. The GFS group had a significantly higher incidence of corneal adverse events, namely corneal edema and Descemet’s membrane striae, than the AFS group (*p* = .035). No significant differences were seen in the remaining complications or adverse events.

**Table 5.** Ocular adverse events, *n* (%).

	AFS	GFS	*p* Value
Corneal opacity	15 (28.3)	26 (18.1)	.035
Corneal edema	9 (17)	17 (31.5)	.08
Descemet’s membrane striae	6 (11.3)	9 (16.7)	.426
Macular edema	1 (1.9)	2 (3.7)	1
Gritty or foreign body sensation	14 (26.4)	15 (27.8)	.874
Dry eye	8 (15.1)	9 (16.7)	.824
Increased lacrimation	5 (9.4)	4 (7.4)	.706
Eye distension	4 (7.5)	8 (14.8)	.234
Aqueous flare	2 (3.8)	5 (9.3)	.449
Subconjunctival haemorrhage	1 (1.9)	0 (0)	.495^a^
Photophobia	2 (3.8)	2 (3.7)	1
Eye itching	4 (7.5)	3 (5.6)	.98
Sticky sensation	0 (0)	1 (1.9)	1^a^

^a^Fisher exact test.

AFS: active-fluidics system; GFS: gravity-fluidics system.

### Subjective perception outcomes

WBS of the AFS group was significantly lower than the GFS group (Table 2). The scores of Cat-PROM5 questionnaire were comparable between the two groups preoperatively, but they were significantly higher in the GFS group at one month postoperatively.

## Discussion

This study evaluated the changes in phacoemulsification caused by the AFS in terms of efficiency, effects, safety and patients’ subjective perceptions in comparison with the GFS. To our knowledge, it is the first comprehensive randomized controlled clinical study aiming at comparison of clinical outcomes between the two systems with a larger sample size. The compliance of the participants enrolled was rigorous, with only three patients lost to follow-up due to the COVID-19 pandemic, which may be attributed to the good trial design and the non-invasive nature of all the examinations.

The results showed that under similar scores of NO and NC, the main outcome CDE was less in the AFS group, though the difference was not significant, and EFU as well. However, another important efficacy indicator TAT was significantly shorter in the AFS group. AFS had an advantage over GFS in maintaining the stable anterior chamber volume and fluid flow, which is expected to increase the followability of the nuclear blocks and thus reducing ineffective manoeuvres [9,27]. Therefore, a reduction in operation time and an increase in efficiency were reached. Some studies have reported that CDE, TAT and EFU were all significantly reduced in AFS than GFS [13,15–17]. The differences in study outcomes were probably due to the fact that most previous studies were based on two phacoemulsifiers like Centurion^®^ and Inifiniti^®^, or two phaco tips like Intrepid Balanced and Kelman [13,16,28]. Furthermore, the same experienced ophthalmic surgeon who conducted the surgery in both groups could reduce unnecessary CDE by controlling the foot pedal, which could be easily found from our lower CDE results when compared with similar studies [15,18,29]. The low CDE status in both groups might explain the non-significant different result.

Similar to the study results of Oh and colleagues [17], there was no significant difference in BCVA between the two groups at one month and three months postoperatively. However, the recovery of BCVA in the early postoperative period (one day and one week) was significantly better in the AFS group. This variation might be strongly related to the higher incidence of corneal adverse events in the GFS group [30,31]. The corneal injuries, which influence the BCVA, like corneal edema and endothelial loss after phacoemulsification have been reported [32,33]. The injuries were attributed to the ultrasound energy and fluid turbulence. Damaged endothelial cells could not regenerate, and the “pump-leak” function, which maintains stromal hydration, is possessed only by healthy endothelium [32,34]. Therefore, it is essential to reduce intraoperative harms, particularly in patients combined with corneal diseases. When the corneal indicators at different time points were analysed, we found that the recovery time for CCT was longer in the GFS group, where a significantly higher CV at one day postoperatively was also recorded. These results suggested that even in cases of comparable CDE and EFU, the application of the AFS could reduce cornea injuries, with shorter postoperative recovery time and better early postoperative BCVA. The phenomenon might be related to the stable fluidics of the AFS, which is able to reduce turbulent flushing of the perfusate from damaging the corneal endothelium [19].

In our study, there was no significant difference in the overall complication rates between the two groups. The incidence of macular edema is similar to previous studies [35,36]. All the three macular edema cases were found in diabetic patients at one month postoperatively. Only one case in the GFS group had obvious macular edema with an increase in CRT of more than 40% [37]. In the remaining two cases, minor changes in macular morphology were observed on OCT and this kind of alteration did not have any impact on the patient's vision acuity.

The effects of phacoemulsification on retinal structure, such as retinal vasculature, CRT and RNFL thickness, have been reported with GFS, while studies with AFS are still lacking [24,38,39]. The VD and PD of superficial macula, which both illustrate the retinal microvascular anatomy but were measured in different ways, were analysed to figure out the vasculature alterations in this trial [24,40]. The angiography we chose had a viewing area of 6 × 6 mm, it was larger compared with 3 × 3 mm and contributed to a more comprehensive assessment [41,42]. Similar to other scholars’ results, the retinal blood flow at the foveal, parafoveal, perifoveal and whole regions was increased after phacoemulsification in both groups [18,20,43]. It has been speculated that the increase might be related to IOP decrease, retinal light exposure increases and intraocular inflammatory factors elevation [20,38,44,45]. It has also been proposed that the perfusion alterations might become an additional benefit of cataract surgery [42,43]. From our results, it took longer for the surgery-induced perfusion changes to fully recover in the GFS group. This discrepancy might be associated with the diverse ways in which these two systems generate and maintain intraoperative irrigation pressure. As a result, less disturbance to retinal vasculature and ocular perfusion pressure was achieved and the time for recovery was shortened. In addition, a significant decrease in IOP after phacoemulsification was also observed, but whether this kind of IOP decrease correlates with retinal blood flow increase requires further study.

The inflammatory response after cataract surgery, such as the release of prostaglandins, leads to a disruption of the blood–retinal barrier (BRB) and thus causing an increase in retinal thickness [46,47]. The RNFL and GCL, changing more rapidly than other retinal layers, are sensitive in evaluating impacts of phacoemulsification [24]. In our study, either the earlier thickening of GCL and RNFL or the significant higher CRT in the GFS group indicate the more quick and severe effects of GFS. The results are probably relevant to the way in which the irrigation pressure is sustained. The unstable intraoperative IOP in the GFS may have contributed to the disruption of the BRB and exacerbated the release of inflammatory factors. And we hypothesize that the thickening in GCL and RNFL is triggered by an increase in blood flow, since the superficial retinal vasculature go precisely in the RNFL and GCL and the vasculature alteration occurred prior to the thickness [24,48].

The fluctuating and rapidly rising IOP in the GFS make patients vulnerable to discomforts such as ophthalmalgia and eye distention, while the subjective perceptions have not been quantitatively evaluated or systematically reported before [4,12]. The results in our study showed that participants in the AFS group had significantly lower pain scores, and the ophthalmalgia was less frequent and less severe. This kind of remarkable improvement in patient comfortableness is sure to be related to the feature of AFS in maintaining a stable intraoperative IOP. In addition, the brief and robust Cat-PROM5 questionnaire was used to assess the impact of cataract on patients' quality of life over the past one month [49,50]. The significantly lower scores in the AFS group suggesting that those who had surgery with the AFS had a better vision-related quality of life. The outcome was probably due to the better optical recovery in the early postoperative period. Based on the above questionnaire and scoring results, it could be asserted that the subjective perceptions of patients were better with the AFS.

There are a few limitations in this study. First, although the sample size has been calculated with caution, it can only indicate the reliability of the CDE and BCVA analysis. For other endpoint parameters included in the study, the reliability is potentially limited by the small sample size, but the conclusions can still be used as a reference. Second, considering the impact of differences between surgeons on the outcomes, we carefully selected a single surgeon to perform all procedures. Although it can avoid obvious confounding factors, it limits the generalizability of the conclusions, which may differ from those of other medical centres. Finally, we only analysed the prognosis within three months after surgery, and the long-term effects of the AFS on cataract patients need to be further studied with a longer follow-up.

In conclusion, compared with the conventional GFS, AFS improves the efficiency and safety of phacoemulsification and reduces the morbidity of complications such as corneal edema. AFS also improves the visual acuity and accelerates the recovery in the early postoperative period. In addition, it helps patients to gain a better subjective perception during and after the surgery. Therefore, AFS is a practical technique that deserves to be widely used to improve the surgical outcome of phacoemulsification.

## Supplementary Material

Supplemental Material

## Data Availability

The data used to support the findings of this study are available from the corresponding author upon request.

## Abbreviations

ACD: anterior chamber depth
AFS: active-fluidics system
AGSPC: active-fluidics versus gravity-fluidics system in phacoemulsification for age-related cataract
AL: axial length
BRB: blood retinal barrier
BSS: balanced salt solution
CCT: central corneal thickness
CDE: cumulative dissipated energy
CRT: central retinal thickness
CV: coefficient of variation
ECD: endothelial cell density
EFU: estimated fluid usage
FAZ: foveal avascular zone
GCL: ganglion cell layer
GFS: gravity-fluidics system
HEX: percentage of hexagonal cells
IOL: intraocular lens
IOP: intraocular pressure
PD: perfusion density
PLA: People's Liberation Army
RNFL: retinal Nerve Fibre Layer
SD: standard deviation
TAT: total aspiration time
VD: vessel density
WBS: Wong-Baker Faces Pain Rating Scale
