# Supplementary material for: A prospective randomized clinical trial of active-fluidics versus gravity-fluidics system in phacoemulsification for age-related cataract (AGSPC)
Source: Ann Med. 2022 Jul 15;54(1):1977–87. doi: 10.1080/07853890.2022.2098375 (PMC9310653; doi:10.1080/07853890.2022.2098375)
Supplement: Supplemental Material [file IANN_A_2098375_SM0053.zip › Supplemental files/Supplementary Table 1.docx]

**Supplementary Table 1. Types of intraocular lens** **implanted, n(%)**

|  | **AFS** | **GFS** | P value |
| --- | --- | --- | --- |
| ZCB00 | 17 (32.1) | 19 (35.2) | 0.734 |
| AcrySof IQ TORIC | 18 (34.0) | 15 (27.8) | 0.489 |
| CT Lucia 601PY | 12 (22.6) | 5 (9.3) | 0.058 |
| HOYA 250 | 3 (5.7) | 9 (16.7) | 0.071 |
| AcrySof IQ | 2 (3.8) | 5 (9.3) | 0.499 |
| AR40e | 1 (1.9) | 1 (1.9) | 1^*^ |

* Fisher exact test.

AFS: active-fluidics system; GFS: gravity-fluidics system.
