# Supplementary material for: A prospective randomized clinical trial of active-fluidics versus gravity-fluidics system in phacoemulsification for age-related cataract (AGSPC)
Source: Ann Med. 2022 Jul 15;54(1):1977–87. doi: 10.1080/07853890.2022.2098375 (PMC9310653; doi:10.1080/07853890.2022.2098375)
Supplement: Supplemental Material [file IANN_A_2098375_SM0053.zip › Supplemental files/Supplementary Table 2.docx]

**Supplementary Table 2. Comparison of endothelial parameters and IOP at different times**

|  | **Preoperative** | **1 Day** | **1 Week** | **1 Month** | **3 Months** |
| --- | --- | --- | --- | --- | --- |
|  |  |  |  |  |  |
| **AFS** |  |  |  |  |  |
| CCT (μm) | 511.32±35.96 | 537.43±40.08 | 528.62±44.30 | 527.58±43.16 | 514.19±37.89 |
| *P value^#^* |  | 0.001 | 0.03 | 0.053^*^ | 0.757^*^ |
| ECD (/mm^2^) | 2604.92±381.63 | 2348.93±524.72 | 2348.89±445.09 | 2402.30±424.44 | 2343.08±453.09 |
| *P value^#^* |  | 0.017^*^ | 0.016^*^ | 0.011 | 0.002 |
| CV (%) | 34.55±9.26 | 35.72±7.51 | 35.96±7.52 | 33.47±7.69 | 32.49±7.03 |
| *P value^#^* |  | 0.241^*^ | 0.238^*^ | 0.704^*^ | 0.333^*^ |
| HEX (%) | 56.55±22.65 | 49.09±21.14 | 54.25±18.89 | 53.17±14.14 | 51.81±15.01 |
| *P value^#^* |  | 0.103^*^ | 0.354^*^ | 0.292^*^ | 0.146^*^ |
| IOP (mmHg) | 13.92±3.28 | 13.79±3.72 | 12.72±3.29 | 11.33±2.71 | 11.32±2.45 |
| *P value^#^* |  | 0.853 | 0.064 | ＜0.001 | ＜0.001 |
| **GFS** |  |  |  |  |  |
| CCT (μm) | 514.11±33.30 | 550.02±46.55 | 527.57±36.71 | 530.28±42.06 | 512.44±34.65 |
| *P value^#^* |  | ＜0.001 | 0.049 | 0.037^*^ | 0.630^*^ |
| ECD (/mm^2^) | 2570.72±382.79 | 2347.54±443.57 | 2265.28±495.05 | 2275.69±418.82 | 2266.59±406.00 |
| *P value^#^* |  | 0.004^*^ | ＜0.001^*^ | ＜0.001^*^ | ＜0.001^*^ |
| CV (%) | 32.26±7.59 | 35.65±7.55 | 33.04±8.94 | 34.20±7.82 | 32.56±7.78 |
| *P value^#^* |  | 0.041^*^ | 0.491^*^ | 0.312^*^ | 0.956^*^ |
| HEX (%) | 52.06±19.86 | 49.06±20.05 | 49.15±21.75 | 47.67±15.07 | 51.00±15.57 |
| *P value^#^* |  | 0.446^*^ | 0.268^*^ | 0.097^*^ | 0.616^*^ |
| IOP (mmHg) | 14.23±3.03 | 13.21±4.03 | 12.36±4.41 | 10.96±2.53 | 10.96±2.19 |
| *P value^#^* |  | 0.047^*^ | ＜0.001^*^ | ＜0.001 | ＜0.001 |

* Mann Whitney U-test. ^#^ P values were calculated by comparing the data at each time point with the preoperative data.

AFS: active-fluidics system, GFS: gravity-fluidics system, CCT: central corneal thickness, ECD: endothelial cell density, CV: coefficient of variation, HEX: percentage of hexagonal cells, IOP: intraocular pressure.
